# Supplementary material for: Cold therapy and pain relief after hot-iron disbudding in dairy calves
Source: PLoS One. 2024 Jul 12;19(7):e0306889. doi: 10.1371/journal.pone.0306889 (PMC11244798; doi:10.1371/journal.pone.0306889)

**S4 Table. Latency post-disbudding model comparison between inclusion and exclusion of 30s ceiling values**

| Model statistic | Model A – ceiling values included | Model B – ceiling values excluded |
| --- | --- | --- |
| Model type | Generalised linear mixed model | Generalised linear mixed model |
| Treatment - t value | -3.22 | -2.11 |
| Treatment - p value (α=0.05) | 0.00129 | 0.0349 |
| Treatment - 95% confidence intervals (CI) | -0.321; -0.0779 | -0.181; -0.00664 |
| Treatment : Expected milk volume interaction - p value (α=0.05) | 0.573 | 0.071 |
| Treatment : Expected milk volume interaction - t value | 0.564 | 1.81 |
| Treatment : Expected milk volume interaction - 95% confidence intervals (CI) | -0.246; 0.446 | -0.0199; 0.483 |
| Number of trials (n) | 588 | 573 |
| Model AIC (Akaike information criterion) | 2766.13 | 1719.47 |
| Model BIC (Bayesian information criterion) | 2083.99 | 1757.10 |

**Supplementary table** – Comparison of latency models including and excluding ceiling values. During the recording of latency during each trial, the trail was ended in the latency to enter the pen exceeded 30 seconds (n=15/588 successful trials). 30 second ceiling values were included in the model (Model A), however a second model was generated which excluded these ceiling values from the analysis (Model B), as this improved the model fit. Model B also yielded a p value for the Treatment:Expected milk volume interaction of 0.071, just short of the α=0.05 threshold.


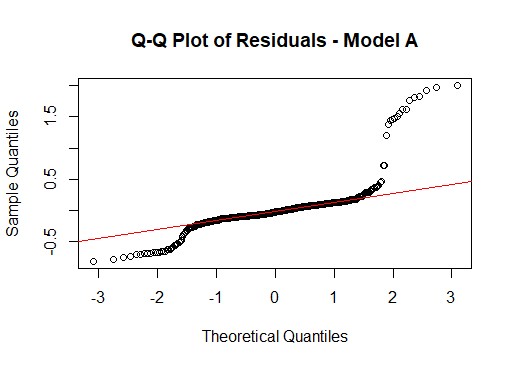


**Data visualisation -** Quantile-Quantile plots of both models for visual comparison.


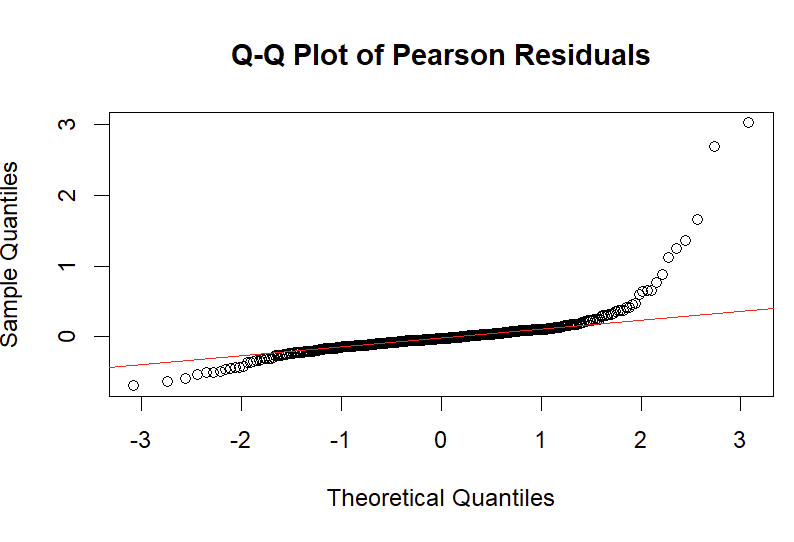


Model B

Model A


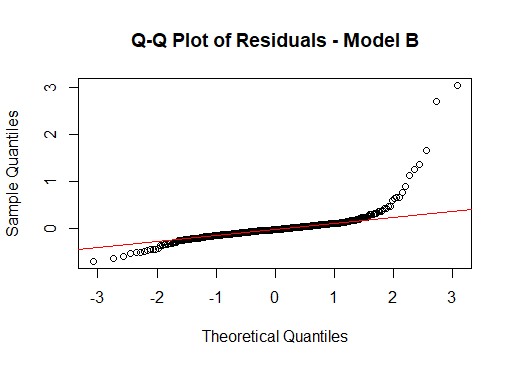

Supplement: S1 Table — (DOCX) [file pone.0306889.s004.docx]
