# Supplementary material for: Cold therapy and pain relief after hot-iron disbudding in dairy calves
Source: PLoS One. 2024 Jul 12;19(7):e0306889. doi: 10.1371/journal.pone.0306889 (PMC11244798; doi:10.1371/journal.pone.0306889)
Supplement: Attachment — Submitted filename: Response to Reviewers.docx [file pone.0306889.s005.docx]

# Authors’ response

We have listed comments from the editor (in red) followed by the measures we took to address them (in black, preceded by >>>), along with manuscript quotations where appropriate. Page/line numbers given in responses refer to the ‘Tracked_changes’ version of the revised manuscript (file name: “Revised Manuscript with Tracked Changes”).

# Responses to Editor:

1. Please ensure that your manuscript meets PLOS ONE's style requirements, including those for file naming. The PLOS ONE style templates can be found at 
<https://journals.plos.org/plosone/s/file?id=wjVg/PLOSOne_formatting_sample_main_body.pdf> and 
<https://journals.plos.org/plosone/s/file?id=ba62/PLOSOne_formatting_sample_title_authors_affiliations.pdf>

>>> Manuscript author affiliations, figures and headings have been edited in accordance with the guidance.

2. To comply with PLOS ONE submissions requirements, in your Methods section, please provide additional information regarding the experiments involving animals and ensure you have included details on methods of anaesthesia and/or analgesia

>>> We are unsure of any additional information that could be provided regarding experimental designs which is explained in length in the Methods section. Methods involved with respect to anaesthesia and analgesia are already detailed:

Line 147-148: “ Calves were administered a subcutaneous NSAID analgesic (meloxicam, 0.5 mgkg-1) and a procaine cornual nerve block (160 mg per horn bud), five minutes prior to disbudding.”

We are happy to address further specific concerns the editor may have.

3. Please note that funding information should not appear in any section or other areas of your manuscript. We will only publish funding information present in the Funding Statement section of the online submission form. Please remove any funding-related text from the manuscript.

>>> The funding statement has been removed from the main manuscript.

4. Thank you for stating the following financial disclosure: 
   "This research was funded by the Animal Welfare Research Network’s Kick-start funding award and the University of Bristol through the start-up research funds attributed to Benjamin Lecorps. "

Please state what role the funders took in the study.  If the funders had no role, please state: "The funders had no role in study design, data collection and analysis, decision to publish, or preparation of the manuscript." 
If this statement is not correct you must amend it as needed. 
Please include this amended Role of Funder statement in your cover letter; we will change the online submission form on your behalf.

>>> The role of funder statement has been included within the funding statement and in the cover letter.

5. In your Data Availability statement, you have not specified where the minimal data set underlying the results described in your manuscript can be found. PLOS defines a study's minimal data set as the underlying data used to reach the conclusions drawn in the manuscript and any additional data required to replicate the reported study findings in their entirety. All PLOS journals require that the minimal data set be made fully available. For more information about our data policy, please see <http://journals.plos.org/plosone/s/data-availability>.

"Upon re-submitting your revised manuscript, please upload your study’s minimal underlying data set as either Supporting Information files or to a stable, public repository and include the relevant URLs, DOIs, or accession numbers within your revised cover letter. For a list of acceptable repositories, please see <http://journals.plos.org/plosone/s/data-availability#loc-recommended-repositories>. Any potentially identifying patient information must be fully anonymized.

Important: If there are ethical or legal restrictions to sharing your data publicly, please explain these restrictions in detail. Please see our guidelines for more information on what we consider unacceptable restrictions to publicly sharing data: <http://journals.plos.org/plosone/s/data-availability#loc-unacceptable-data-access-restrictions>. Note that it is not acceptable for the authors to be the sole named individuals responsible for ensuring data access.

We will update your Data Availability statement to reflect the information you provide in your cover letter.

>>> The minimal underlying dataset was uploaded as Supporting Information file (S1) (pg 18) when submitted initially and as indicated during the submission process. We now make this clear in the cover letter as well.

6. Please include captions for your Supporting Information files at the end of your manuscript, and update any in-text citations to match accordingly. Please see our Supporting Information guidelines for more information: <http://journals.plos.org/plosone/s/supporting-information>.

>>> Supporting Information has been updated accordingly (pg18).

Additional Editor Comments:

Before this manuscript can be sent out for peer-review, please revise the manuscript addressing the following:
- Using correct epidemiological terms, define the type of study design utilized. Then, follow the relevant reporting guidelines. Currently, many essential information that must be reported is missing. When resubmitting, please include the checklist of the appropriate reporting guidelines outlining where in the manuscript you are reporting each item.

>>> As this is not an epidemiological study (but rather an experimental one), we are unsure on how best to describe the study design. Can the editor be more specific about the ‘many essential information’ required that would be missing? This is not the first time we submit to PLOS One and this manuscript is not less complete than any of our previous ones. Please note that we have included in our submission a copy of a completed ARRIVE Essential 10 guideline, with line number corresponding to the updated manuscript.

- Provide the specific details of the sample size calculation. Simply stating "we used previous peer-reviewed publications with similar methodologies as a basis for our sample size calculation" is inadequate.

>>> We have addressed concerns regarding the calculation/justification of our sample size. Because no previous studies combined approach avoidance and conditioned place avoidance tests before, nor assessed the effect of cold therapy to study pain in this animal species or in any other, we could not directly use previous effect sizes for *a priori* power calculations. This means we had to rely on estimated effect sizes.

Line 82: “Using the *pwr* package in R [33], a minimum sample size of 17 individuals was calculated for a statistical power of 0.8, a small effect size (f2=0.02) and a significance level of 0.05 using linear models for our two main variables (latency and post-drinking contact duration in the approach-avoidance test). A similar power calculation for t-test found that 15 individuals would be necessary to detect strong effect size, consistent with previous studies...”

[Note: HTML markup is below. Please do not edit.]

[NOTE: If reviewer comments were submitted as an attachment file, they will be attached to this email and accessible via the submission site. Please log into your account, locate the manuscript record, and check for the action link "View Attachments". If this link does not appear, there are no attachment files.]

While revising your submission, please upload your figure files to the Preflight Analysis and Conversion Engine (PACE) digital diagnostic tool, <https://pacev2.apexcovantage.com/>. PACE helps ensure that figures meet PLOS requirements. To use PACE, you must first register as a user. Registration is free. Then, login and navigate to the UPLOAD tab, where you will find detailed instructions on how to use the tool. If you encounter any issues or have any questions when using PACE, please email PLOS at [figures@plos.org](mailto:figures@plos.org). Please note that Supporting Information files do not need this step.

>>> all figures have been run through PACE and are in a .tif format.
