# Supplementary material for: Cold therapy and pain relief after hot-iron disbudding in dairy calves
Source: PLoS One. 2024 Jul 12;19(7):e0306889. doi: 10.1371/journal.pone.0306889 (PMC11244798; doi:10.1371/journal.pone.0306889)
Supplement: Attachment — Submitted filename: Response to Reviewers.docx [file pone.0306889.s006.docx]

**Line numbers in the following responses to comments refer to the revised manuscript without tracked changes.**

**Editor Comments:**

In the manuscript, please specify the type of study using adequate terminology (e.g., case-control, cross-sectional, cohort, randomized control trial, etc.)
>>> We have included this in our introduction (line 71). it now reads:

“For this we conducted an experimental control trial, using a methodology combining an approach-aversion test and a conditioned place preference (CPP) procedure.”

**Reviewers' comments:**

**Reviewer #1:**

This paper evaluates the use of "cold therapy" as a mechanism to reduce pain associated with dehorning. There are several challenges associated with the description of the study that makes it difficult to interpret the results. Furthermore, disbudding was completed very late and many different breeds were used. The practical relevance of producers using cold therapy is also a concern.

>>> Thanks for your comments. We designed this experiment as a proof of concept. Our aims were to assess the possibility of providing pain relief using cold therapy so that we can then use calves’ preference or willingness to work for this to assess for how long calves are in pain following disbudding. This knowledge will then inform pain mitigation strategies. Of course, an application of cold therapy to provide pain relief post-disbudding may derive from this work but this was not the primary purpose of this paper.

Specific comments can be found below:
Line 20: Please clarify the type of analgesia provided

>>> abstract edited to include meloxicam – line 20 now reads “Calves were then disbudded under local anaesthesia (procaine) and analgesia (meloxicam) ”.

Line 21: It is unclear from the abstract how frequently the "cool packs" were changed or how they were placed on the calf's head and for how long. These seem like critical details to assess the practicality of this approach working.

>>> the abstract has been reworded to address the concerns raised. We are however restricted with abstract word limits, and the details regarding the nature of pack contact are elaborated upon in the methodology. The abstract now reads (line 18-20): “Calves were habituated to the manual placement of a cool or ambient pack on their forehead for a short duration simultaneous to milk reward consumption, prior to disbudding.”

Line 23 to 24: The calves were housed in completely separate pens when the treatments were being applied? This seems like it could introduce substantial bias as it would be impossible to control for the impact of pen or calves within the pen.

>>> Sorry for the confusion. Calves were housed for the entire duration of the study in a familiar hutch (described in the ‘animals and housing’ section). During experimental sessions, calves were temporarily moved to the experimental arena (figure 1) – this experimental arena was the same for every calf tested. Each calf received the two different treatments (cool pack application and ambient temperature control pack application) in different sides of the colour-coded experimental pen (figure 1), but the pseudorandomised counter-balancing of treatment and colour allowed us to control for the impact of pen side (i.e. half the calves receive the cool pack on the blue side and ambient on the white, the other half of the calves this is reversed). All experiments were conducted with calves one at a time, so no influence of other calves in the experimental pen needs to be considered.

To try and make this clearer in the abstract, lines 21-23 have been changed to: “Individual calves were consistently exposed to either cool or ambient packs in different halves of a two-sided experimental pen, allowing for the testing of approach-aversion and conditioned place preference”

Line 39 to 41: I think it is important to note that the use of pain mitigation is low in some countries. For example, in Canada, the overwhelming majority of producers use pain control (it is a requirement), whereas in the US, most use pain control as well. This brings up another point that is worth discussing in the discussion of this paper. If few producers are using pain control, how will we convince them to use cold therapy which would seemingly be harder to manage?

>>> This is an interesting comment regarding the promotion of changes in pain-management practices. As explained in response to a previous comment, the aim of this study was not to explore the applicability of cold therapy to mitigate pain associated with cold therapy on a farm.

Line 56 to 58: How was it determined to be beneficial? Through preference testing?

>>> This paragraph has now been reworded for clarity. It now reads (line 55-61):

“Conditioned place preference (CPP) tests – a commonly used paradigm to assess the rewarding effects of a specific stimulus or experience as a result of prior conditioning [28] – have also indicated that post-procedural pain and its associated emotional impact are remembered by calves [7]. Using the CPP paradigm, calves have also shown preference for the provision of analgesia with disbudding [29], as well as prolonged pain control (i.e., in the form of a local anaesthetic injection) up to 20 days after disbudding when compared to sham disbudded controls [14], suggesting that they were still in pain.”

Line 85 to 86: Above, it mentions few have done this type of work making it difficult to calculate; however, here it suggests previous studies are done? Basing sample size on simply what other studies have enrolled is likely inappropriate and should be deleted. It is different to use effect sizes found in the studies to come up with a calculation.

>>> Perhaps the reference to ‘previous studies’ has been misleading here – this specific methodology is novel and as such power calculations could not be drawn directly from previous effect sizes. Instead, we used the sample size and effect size of previous experiments conducted on calf responses to pain that use the approach-avoidance paradigm, as this is the closest we are able to get to direct experimental comparisons. Whilst not ideal, this was the optimal approach to determining a sample size when no similar work has been conducted.

In light of this feedback, we have amended this sentence (line 96-97): “ …consistent with previous studies that have also used an approach-avoidance paradigm to investigate calf response to painful stimuli [29], [36]”

Line 87 to 88: I assume that breeds would not be balanced between groups making it difficult to control for the likely effect that breed could have on the testing done in this study. How was that controlled for? If not controlled for, this should be noted as a limitation.

>>> This study did not aim to assess whether calves of different breed would react to disbudding in different ways. Although we agree no conclusions can be drawn on breed effect, we disagree that having multiple breeds is a weakness, especially when considering that we used within-calf comparisons. In contrast, this is likely to render our results more applicable and translatable to what happens on farms (given that crossbreeds are typically disbudded). We also call the reviewer’s attention to the risks associated with over-standardization and the benefits to introduce controlled variation in experimental designs (see the work of Hanno Wurbel’s team, for example: https://doi.org/10.1038/s41583-020-0370-7). The methodology of our experiment as a within-subject design means breed was controlled for in this manner.

Line 88: When were calves normally weaned at this facility? Why was disbudding completed so late? Earlier disbudding would reduce inflammation and the size of the lesion. Likely important to preface the findings with the dehorning time in mind.

>>> Calves are typically weaned at 12 weeks of age at this farm. We can only speculate about farm staff’s decision and rationale, but we think it was related to the perception that young calves are more vulnerable and therefore should not be disbudded. As researchers, we had limited control over when disbudding was carried out for this experiment. If that can be of any reassurance, calves’ horns were not developed, and the procedure could still be considered disbudding. Furthermore, Marquette et al. (2021) (https://doi.org/10.1186/s13620-021-00196-0) suggests horn size is more relevant than age regarding recommendations for time of disbudding, and Mirra et al. (2018) (https://doi.org/10.1016/j.physbeh.2017.11.031) shows that age at disbudding does not affect pain sensitivity. Considering there is no clear-cut distinction between disbudding and dehorning and that age is not the only factor at play, we would prefer to keep the language consistent here and avoid bringing some confusion by talking about disbudding vs dehorning.

We have added the following paragraph to the discussion section to clarify the concerns raised (lines 367-375):

“Calves were disbudded at a mean age of 58 days (approximately 8 weeks). Although most recommendations cite 8 weeks as an upper limit for calf disbudding, this is not based on empirical evidence, instead based on the physiological timeline of underlying periosteum development [42]. Evidence suggests that horn size, rather than age, should be used primarily when making recommendations for time of disbudding, as this accounts for variability in breed and sex at a given age [43]. Equally, disbudding too young has shown to have negative consequences on calf pain sensitivity in the weeks after disbudding [3], [21]. Whilst we were limited in our ability to control age at disbudding, all calves enrolled in the study were deemed suitable for disbudding by a qualified veterinarian.”

Line 92: Additional information is needed on the feeding program provided ahead of time.

>>> The feeding regime prior to the experiment with the calves was identical to what was received during the experiment (*ad libitum* access to water, grain and straw, plus 7.5L milk per day). For clarification, the section under ‘Animals and housing’ now reads (line 101-105):

“Calves were given *ad libitum* access to water, grain, and straw, as well as a total of 7.5L of milk replacer per day during the experimental sessions (Sprint Plus 50, Bridgmans Farm Direct; 150gL^-1^), split equally between experimental sessions in the morning (0800 h) and afternoon (1600 h). This is identical to the feeding program delivered to calves prior to experimentation.”

Line 93: 190 g of milk replacer per L is very concentrated. Is this correct?

>>> This was a typo. The concentration was 150gL^-1^, and this has been corrected in the manuscript.

Line 94 to 95: Were groups made up to contain both treatment groups?

>>> Every calf experienced both treatments, alternating between cool and ambient pack application on different sides of the pen. This is explained in the ‘experimental procedure’ section, and has been elaborated on in a revised figure 2 (below).

Line 110: How was this temperature maintained?

>>> Cold packs were kept in a fridge, maintained at 4ᵒC until use. Each pack was swapped for another identical pack at 4ᵒC after each experimental session with a calf. Using an infrared thermometer, we confirmed that over the duration of a session (a maximum of 20 minutes), cool pack temperatures only increased by a maximum of 1ᵒC.

To clarify, we have added a statement to this effect (line 127-129): “Cool pack temperatures were monitored using an infrared thermometer (SOVARCATE HS960D), confirming that pack temperatures did not change by more than 1ᵒC during a given session.”

Line 118: Was the cold pack only applied for 20 min (or seems like 40 min per day for 6 days) and no longer? How effective is short application of cold packs in controlling inflammation? Is there any data or studies that could be cited here to provide insight into why this threshold was chosen?

>>> This comment is a misunderstanding of our methodology, for which we apologise. Packs were not consistently applied for 20 minute durations, but rather *only* when calves were drinking milk in a given trial. In a session that typically lasts for a maximum of 20 minutes, 6 trials take place, but the exact duration that time the pack is applied is variable, as it depends on how long the calf chose to suckle in each trial. Furthermore, our intention in this study was not to use cold therapy for its anti-inflammatory properties, but rather for its instantaneous pain relief effects (compare this to the immediate pain relief in humans experienced from cold running water or the application of a cool pack on a burn).

To help avoid this misunderstanding, we have changed a sentence in the introduction, which now reads (lines 63-68): ““Whilst less regularly used in animals for analgesic purposes, cold therapy is suggested as “…an effective analgesic tool for acute pain management” [32], and appears suitable to cautery disbudding given the nature of the injury associated with the procedure. With running water being likely impractical for the disbudded wound [33], cold spray as a pre-disbudding pain mitigation strategy has been trialled previously in calves indicating an analgesic effect [34].”

We have also made a small edit to the introduction to emphasise that we are investigating instantaneous pain relief, rather than prolonged anti-inflammatory relief, so the final paragraph now starts (69-71): “The aim of our study was to investigate whether cold therapy, through the manual application of a cool pack to the disbudding wound, would be able to provide instantaneous pain relief on the days following the procedure.”

Line 125: How was this milk prepared (temperature and g/L)? Was it the same milk replacer? Was time relative to last milk replacer meal controlled for? i.e., if a calf was fed milk at 8 am, was it tested at 8:30? Similarly, if a calf was fed at 8 would it be tested at 13:30? This would have a major impact on milk consumption.

>>> We added some information (line 105-108):

“Milk replacer was mixed with warm water (45ᵒC) as stated by the provider. Experiments were conducted at the meal times (8am and 4pm), so when calves did not receive their full milk allowance (3.75L per meal) during an experimental session, they were fed the remaining amount just after the session ended.”

Line 147 to 148: Pleas add in the brand of meloxicam and procaine used.

>>> The brand names have been added as requested. the sentence now reads (lines 171-172):

“Calves were administered a subcutaneous meloxicam analgesic (Metacam, 0.5 mgkg-1) and a procaine cornual nerve block (Procamidor Duo,160 mg per horn bud), five minutes prior to disbudding.”

Line 155: This is confusing. 3.75 L were consumed per session? I assumed it went down as highlighted in Line 151 to 153. Please clarify

>>> Sorry for the confusion. See previous comments for clarification.

Line 164 to 165: Why were they excluded?

>>> Trials were ended immediately if latencies exceeded 30s. Although there is always a cost in applying cut-off thresholds, 30s is often used in studies where calves have to make a choice (e.g. Lecorps et al., 2018). We subsequently felt exclusion of ≥30s latencies was the most appropriate approach. Whilst this reduced our data size slightly and prevented us from capturing extremely aversive calf responses, including ≥30s latencies may have had a disproportionate effect in our models. We therefore chose to exclude these latencies from the model. To this effect, the section now reads (line 188-192):

“If the latency for a given trial exceeded 30s, the trial was ended and the calf returned to the lobby area, to commence the next trial, consistent with previous studies [27]. Latencies exceeding 30s were excluded from subsequent statistical analysis (21/588 trials excluded). Whilst cut-off thresholds prevent the capture of extremely aversive calf responses, the exclusion of these latencies ensured our models were not disproportionately influenced by extreme values.

Line 171: Why are these excluded?

We excluded calves that took greater than 60s to drink for a similar reason – given the rate of consumptions noted previously, we considered 60s to consume the reward to be a sufficiently long time to allow for consumption. A cut-off time was necessary to maintain experimental timings, particularly when multiple calves per session needed to be tested sequentially. Again, data points ≥60s drinking duration were excluded to eliminate the effects of extreme values in our models, therefore taking a conservative approach.

Line 191: How were these models evaluated to ensure model fit?

>>> Models were initially assessed for normality and heteroscedasticity through analysis of the residuals. The choice of normal or non-normal distribution models (LMM vs GLMM with gamma distribution) was made based on residual analysis, and comparison of model fit with AIC/BIC criteria. The inclusion/exclusion of each random effect was also analysed stepwise through comparisons of these criteria.

Line 233: Please provide information about the comparability of the two treatment groups including age at disbudding and breed to ensure comparable between groups.

>>> As all calves enrolled in this study experienced both treatment groups (“within-subjects design”), there is no need to account for age or breed differences.

Line 241: Pack temperature or treatment group?

>>> We referred to pack temperature; referring to treatment groups for a within-subjects design would not be appropriate.

Line 259: Was coloured controlled for in the model as well?

>>> Yes – colour (representing each half of the test pen) was included in the model (see statistical analysis section (lines 229-231): “Mixed models were then constructed for each outcome variable, which included milk volume per trial, colour (‘Blue’ vs ‘White’), treatment (‘Cool’ vs ‘Ambient’) and the 2-way interaction between milk volume per trial and treatment as fixed effects.”)

Line 300 to 301: What does "near-significant" mean? This is also not in the results as far as I can tell.

>>> We agree that ‘near-significant’ may be confusing. As such, we have changed this to ‘tendency’, now reading (line 328-330): “The finding of a non-significant tendency for calves to come faster, as expected milk volume decreased, to the bucket associated with the cool pack but not to that associated with the ambient pack agrees with…”. This result is referred to in the results section (line 276-277): “…the interaction between expected milk volume and treatment in the latency model fell just short of the significance level (t = 1.805, P = 0.0711, 95% CI: -0.020, 0.483).”

Line 314 to 317: I find this very confusing. Could a different word be used instead of trial? When were the calves disbudded and how did time relative to disbudding come into play?

Yes, differentiating between day, session and trial could be confusing. We kept the term to remain consistent with the text, but we have amended fig.2 and its caption to improve clarity.

figure 2 caption now reads:

Fig 2: Timeline illustrating the experimental procedure.

The top graph illustrates the volume of milk given to calves per trial in each experimental session. Numbers on the x-axis correspond to each experimental session. Session 1-4 represent the training and habituation phase. Session 5 corresponds to the pre-disbudding baseline session. Disbudding occurs between session 5 and 6. Session 6-11 correspond to the testing phase with progressively decreasing amount of milk rewards. The conditioned place preference test occurred immediately following session 11. Each session took approximately 20 minutes per calf. The layout of each session is also illustrated, with each session consisting of 6 separate trials, alternating between a cool pack (blue box) and ambient (red box). In this example, the cold pack was associated with the blue test pen and the ambient pack associated with the white test pen. These pen colour/treatment paired associations, as well as the pack temperature of the first trial, were pseudorandomised and allocated evenly across participants.

**Reviewer #2:**

This study uses approach-avoidance as well as Conditioned place preference to evaluate the effect of a cool pack on post disbudded calves. 15 calves were enrolled prior to the disbudding procedure and habituated and conditioned to reception of a cool or ambient pack while drinking milk. Calves were disbudded with local and systemic anaesthesia and analgesia. The authors found that calves had a reduced latency to approach when a cool pack was offered vs. an ambient temp pack, but that calves did not display a preference to the pen where they received the cool pack.

This is a very interesting study that pairs two tests to evaluate the emotional component of pain/pain relief. The study is well written.

Authors: thank you for the positive comments!

Comments:
L41. Is disbudding a procedure that has to be performed by a veterinarian in the UK? Is that why “workload” is cited as a reason to not provide pain mitigation? Please clarify.
>>> Whilst it is strongly encouraged that veterinarians perform disbudding, it is not illegal nor unusual for a non-veterinarian to perform disbudding in the UK. To clarify this point, we have removed reference to ‘veterinarian’. The sentence now reads (line 42-43):

“with cost and workload cited as primary factors in a farmer’s decision to forgo using pain relief [15], [16], [17]”

L58. In that study, calves at 20d all still had necrotic buds, so is it still pain? What about sensitivity? Itchiness due to healing epithelial tissue?
>>> Fair enough, although we still think pain is the most likely of all hypotheses. We now say here (line 60-61) “suggesting that they were still in pain” to encompass the possibility that pain may not be the main or only driver of this response.

L88. These calves are quite old to disbud (with most (Holstein) recommendations begin to disbud younger than 4 weeks of age). Why was the procedure done so late?

>>> We agree with the reviewer, but we had limited control over disbudding age as this was current farm practice. We share similar concerns although recent research does not seem to support an effect of age on disbudding pain (see Mirra et al., 2018).

please also see response to Reviewer 1’s comment regarding age of weaning and disbudding:

>>> Calves are typically weaned at 12 weeks of age at this farm. We can only speculate about farm staff’s decision and rationale, but we think it was related to the perception that young calves are more vulnerable and therefore should not be disbudded. As researchers, we had limited control over when disbudding was carried out for this experiment. If that can be of any reassurance, calves’ horns were not developed, and the procedure could still be considered disbudding. Furthermore, Marquette et al. (2021) (https://doi.org/10.1186/s13620-021-00196-0) suggests horn size is more relevant than age regarding recommendations for time of disbudding, and Mirra et al. (2018) (https://doi.org/10.1016/j.physbeh.2017.11.031) shows that age at disbudding does not affect pain sensitivity. Considering there is no clear-cut distinction between disbudding and dehorning and that age is not the only factor at play, we would prefer to keep the language consistent here and avoid bringing some confusion by talking about disbudding vs dehorning.

We have added the following paragraph to the discussion section to clarify the concerns raised (line 367-375):

“Calves were disbudded at a mean age of 58 days (approximately 8 weeks). Although most recommendations cite 8 weeks as an upper limit for calf disbudding, this is not based on empirical evidence, instead based on the physiological timeline of underlying periosteum development [42]. Evidence suggests that horn size, rather than age, should be used primarily when making recommendations for time of disbudding, as this accounts for variability in breed and sex at a given age [43]. Equally, disbudding too young has shown to have negative consequences on calf pain sensitivity in the weeks after disbudding [3], [21]. Whilst we were limited in our ability to control age at disbudding, all calves enrolled in the study displayed visible horn buds and were deemed suitable for disbudding by a qualified veterinarian.”

L128: Videos didn’t work for me, which was disappointing!
>>> We apologise for this. We will make sure videos are resubmitted in a viewable format for this round of revision.

L134: So each calf received each pack/color combination (which stayed the same so if calf 1 had blue/cold they always got blue/cold)? Thus if that calf wanted the cold pack after disbudding, they would have to go to blue? Why not apply the cold pack to the calf post disbudding for some period of time paired to color? Couldn’t it be that cold on a “normal” calf head isn’t the same as cold on a disbudded head (soothing the inflammation, for example) and that you would have had a stronger response? Please clarify in the methods.

>>> Yes, this is correct – each calf received the same colour/pack combination, so that calves may associate pack with a specific side of the test pen, allowing us to test for conditioned place preference at the end of the experimental week. Regarding your second point about applying the cold pack to the calf head post disbudding, our expectation was that the initial cost of the cool pack (before disbudding) would change given the rewarding properties of the cool pack.

To make this expectation clearer, we added the following to the introduction (line 77-79):

“Our assumption was that the inherent value of the cool and ambient packs would change after disbudding from mildly aversive to aversive for the ambient pack and from mildly aversive to rewarding (because of pain relief) for the cool pack.”

L343. Yes, would have been very interesting to give the calf the choice (milk meal paired with color/pack) but that would be much more difficult to habituate/train?

>>> Agreed. This is something we are considering for future experiments.

L346. Consider adding some discussion of calf age and how age (and bud size) might impact the calf response to this type of treatment. Would suspect that younger calves (shorter burn time) might not respond the same way? Any more information available on technique used in this study? Time of procedure (how long to burn?)?

>>> Please see our response to a previous comment on age at disbudding.

Figure 4. Confused by the Trial 1/Trial 2/Trial 3…..what are these? There is no reference to these three trials anywhere else in the manuscript (that I can find). Please clarify.

>>> What we meant by trials was explained in the experimental procedure section. To try and improve clarity, we have modified figure 2 to include information about the 6 separate trials per session (see image in response to reviewer 1).

**Reviewer #3:**

I commend the authors for their creative approach to attempt to offer calves autonomy and additional pain relief given the long-lasting pain from disbudding.

>>> Thank you for the positive comments!

I appreciate the acknowledgement of the limitations for this study, such as the motivation for milk influencing the latency to respond in Trial 1. However, there were several conceptual areas that I found concerning or where my interpretation is that additional information would be useful.

>>> We address your comments below.

1.) The use of an approach/aversion paradigm and conditioned place preference/aversion are not consistently described throughout the manuscript. I would benefit from some clarification and consistency.
o Ln 67 lays out the test as an approach-aversion test, while the predictions do not specify which aspect of the treatments would be aversive (ln 70-72).

>>> Agreed, we now specify this in the introduction, which now reads (line 71-83):

“For this we conducted an experimental control trial, using a methodology combining an approach-aversion test and a conditioned place preference (CPP) procedure. Calves were offered a milk reward which they could access at the ‘price’ of having a gel pack (at cold or ambient temperature) in contact with their wound site. As previously noted, calves display increased sensitivity of the injury area after disbudding [35]. By presenting calves a conflicting choice between events of positive (milk reward) and negative (contact pain) valences, the difference in motivational trade-offs between the two pack temperatures could inform us on the effectiveness of cold-therapy as a pain mitigation strategy following disbudding. We expected that, following disbudding, calves would approach the milk reward associated with application of the cool pack quicker and spend more time in contact with the cool pack, in comparison with the aversive ambient pack. In the CPP test, we expected calves to spend a greater proportion of time in the coloured pen associated with the cool pack."

o In Ede et al 2018 (10.1038/s41598-018-27669-7), the treatment was something the authors predicted that the calves would avoid (injection pain), thus naming it an approach/avoid paradigm in their work. As far as I can tell, in the current study, the prediction was that the calves would like the cold (ln 70-72), thus it becomes an approach/approach paradigm, which is more difficult to untangle in terms of what it means to the calf than what Ede et al 2018 did. Consider discussing this.

>>> This is a good point. However, we think our results clearly show that placing a pack on the disbudding wound was aversive (less so for cold) although not enough to prevent most calves from coming to drink even when low milk quantities were provided.

o It would be reasonable to predict that calves would find either of the packs aversive given the

increased sensitivity following disbudding. This is touched on this briefly in ln 310. If you choose to continue describing the test as an approach-aversion test, I would benefit from some context for the aversive component in the introduction. In addition to references 12, 20, and 21, you may consider discussing other literature that provides evidence of sensitivity to touch following disbudding, to support the aversive component of your test (e.g.: 10.3168/jds.2021-21552; 10.3168/jds.2023-23238; 10.3168/jds.2009-2813).

>>> Thank you for this, we now include more information in the introduction, see previous response. We also note at this point that ‘approach-avoidance’ was accidentally used in the manuscript, rather than ‘approach-aversion’. We have changed all mentions of the incorrect term accordingly.

o Ln 177-181 begins by describing the use of a conditioned place preference test, but ends by stating calves were tested for aversion. Ln 281-284 and Figure 5 present the preference of calves for the cold pack, but do not report any conditioned place aversion. Please clarify if conditioned place aversion was also tested or observed.

>>> This was a mistake. Preference and avoidance really are two sides of the same coin here as calves could only spend time in one or the other pen (in contrast with Ede et al where calves could also spend time in the ‘neutral’ zone connecting the two pens). Nevertheless to remain consistent and avoid confusion, we have removed the use of conditioned place aversion, so only conditioned place preference is included in the wording. The data collection section now reads (line 208-209): “Calves were fed at least 10 minutes before being tested for conditioned place preference. This was done to prevent calves from seeking food in the arena during the test.”

2.) The conclusions drawn are overstated and do not seem supported by the approach or data presented
o Ln 350-351: I do not see evidence presented in the current paper of the analgesic effects of cold therapy for calves. Rather, calves seek a milk reward faster with a cold pack is applied to their head compared to an ambient-temperature pack.

o Ln 26-27, 306: I do not see evidence that “suggests the cold therapy provides some form of pain relief post-disbudding” Rather, calves seek a milk reward faster with a cold pack is applied to their head compared to an ambient-temperature pack. Pain relieving characteristics were not evaluated.

>>> Given the overwhelming evidence that cold therapy provides pain relief in humans follow burn injuries, coupled with the observed differences between pre- and post- disbudding, we believe that the results of this experiment are best explained by the immediate pain-relieving property of cold therapy on the disbudding wound. We do however accept the limitations of this study, and we have amend the last paragraph of the discussion. It now reads (line 389-391): “Further research to understand the benefit of post-procedural cold therapy in calves is necessary, possibly through the autonomous application of cool packs or other cooling mechanisms to limit human involvement and biases.”

o Ln 346: Further work is needed to understand possible benefits of cold therapy, rather than “confirm”.

>>> this change has been made – see response to earlier comment.

o As the cold therapy was tied to a milk reward, it is difficult to disentangle the effects of the cold therapy from their motivation for milk. With the current methods, calves will likely approach the packs unless they are not hungry, which seems unlikely at this plane of nutrition and age of the calves. I would benefit from further discussion of the implications of this context when assessing the results.

>>> As the random effect of individual calf differences were accounted for in our model, variability in absolute motivation for milk between individual calves was not important here, only the relative change in individual calf motivation pre- and post- disbudding. This applies to both treatments in a similar way.

3.) Use of the milk reward in conjunction with the compress.
o In Ln 92, it seems calves being provided their milk meals only in the arena? Was there any milk meals provided outside of the training or test sessions?

>>> Calves received their daily amount of milk through a) the experimental sessions (AM and PM) and b) immediately after a session, any milk that they did not consume during the experiment (to ensure a total of 7.5L per day is consumed). Remaining milk, provided after the experimental session, was given to them back in their hutches, not in the experimental test pens.

o Ln 154-155 was the remaining milk replacer fed in different location? Was this changed in any way as the reward size changed?

>>>see the response above. This was not changed as reward size decreased.

o Ln 187-188 given the ages, were any calves being weaned during the trial?

**>>>** No calves were weaned or being weaned during the trial.

o The drinking speed seems rather fast (0.5L/60 sec; ln 151, 171), I would benefit from some contextualization of these speeds, how they compare to other literature, and biologically normal drinking behavior.

>>> We disagree that consuming 0.5L in 60 seconds is fast. Calves in the experiment regularly consumed 0.5L of milk within 60 seconds, some in substantially less time (see S1 – ‘Drinking_Duration_scaled’ column for sL^-1^). Perttu et al (2023, <https://doi.org/10.3168/jds.2022-22043>) found drinking speeds with automated feeders at 0.811L/60 sec, whilst Braun et al (2022, https://doi.org/10.1186/s12917-022-03280-x ) found calves at 5 weeks of age achieving 1L/60 sec. We only ever compared drinking durations pre- and post- disbudding as a proxy for motivation to drink, and therefore the absolute drinking speeds were not of too much concern for this experiment, especially as they fit within existing expectations of calf drinking speeds. To address this point, we have included the following sentence under the ‘data collection’ section (line 198-200): “The consumption of 0.5L of milk within 60s is well within previously reported calf drinking rates [37], [38] and was therefore deemed an appropriate cut-off duration.”

o What are the possible implications of initiating the suckling response to then end a training session and restart? For more context about how milk stimulates sucking, see, for example, de Passillé 2001, Sucking motivation and related problems in calves, 10.1016/s0168-1591(01)00108-3

>>> We have used this type of experimental design in the past (where calves receive multiple small milk rewards) in many studies (judgment bias tests, approach avoidance, reward sensitivity etc.) and have not noticed any negative effect on calves. In the current experiment calves always drank their full allowance and if anything our repeated small quantities of milk may have induced more salivation which is known to have positive effects on GI health.

4.) What do we know about cold therapy?
o I would benefit from additional information on the use of cold therapy.
o How long is cold therapy needed to receive the benefits of pain relief? Is it possible to provide context of timing in humans and other species?
o I appreciate that citations 29 and 30 are reviews of this topic, is there specific evidence that can be pulled to help provide additional context for this pain relief strategy?

>>> Our study is the first to look at the application of cold therapy through contact with the disbudding wound in calves. Cold therapy through cold water is one of the first line treatments for burns in animals as well as humans. While literature on its application for analgesia in animals is scant, a recent review in 2020 concludes that “Based on the human literature and limited evidence in veterinary medicine, cold therapy is an effective analgesic tool for acute pain management – it is medically useful, scientifically sound and readily available” (Wright et al, 2020 - 10.1111/jsap.13130). Whilst most typically applied through cold running water, we felt this to be difficult to utilise in the calf context, opting instead for cold packs. To provide context, we have amended the introduction – it now reads (63-68):

“Whilst less regularly used in animals for analgesic purposes, cold therapy is suggested as “…an effective analgesic tool for acute pain management” [32], and appears suitable to cautery disbudding given the nature of the injury associated with the procedure. With running water being likely impractical for the disbudded wound [33], cold spray as a pre-disbudding pain mitigation strategy has been trialled previously in calves indicating an analgesic effect [34].”

o Is there evidence that the cold therapy actually cooled the wounds? Surface temperature of the calf’s head at the start vs after pack application?
>>> We did not measure the temperature of the disbudded wound before and after application. As this experiment was interested in the instantaneous pain relief provided by cold contact (rather the anti-inflammatory effects of prolonged cooling), the surface temperature of the wound after pack application was not of relevance here. However, we can confirm that the application of the cool pack cooled down our hands!

5.) Why were the calves/trials where milk drinking persisted for longer than one minute excluded?
o What was the number of calves effected by each exclusion of data? How did those relate to treatment (e.g., Ln 165, 171)?

>>> Trials were ended immediately if latencies exceeded 30s. Although there is always a cost in applying cut-off thresholds, 30s is often used in studies where calves have to make a choice (e.g. Lecorps et al., 2018). We subsequently felt exclusion of ≥30s latencies was the most appropriate approach. Whilst this reduced our data size slightly and prevented us from capturing extremely aversive calf responses, including ≥30s latencies may have had a disproportionate effect in our models, making detection of differences between groups difficult. We therefore chose to exclude these latencies from the model. To this effect, the section now reads (line 187-192):

“If the latency for a given trial exceeded 30s, the trial was ended and the calf returned to the lobby area, to commence the next trial, consistent with previous studies [27]. Latencies exceeding 30s were excluded from subsequent statistical analysis (21/588 trials excluded). Whilst cut-off thresholds prevent the capture of extremely aversive calf responses, the exclusion these latencies ensured our models were not disproportionately influences by extreme values.”

We excluded calves that took greater than 60s to drink for a similar reason – given the rate of consumptions noted previously, we considered 60s to consume the reward to be a sufficiently long time to allow for consumption. A cut-off time was necessary to maintain experimental timings, particularly when multiple calves per session needed to be tested sequentially. Again, data point ≥60s drinking duration were excluded to eliminate the effects of extreme values in our models, therefore taking a conservative approach.

o The exclusion of these trials or limiting the compress contact to ~2 minutes seems to assume that the benefits from the cold are less than 60 seconds. Is there evidence to support this assumption?
>>> As noted in responses above, this study was interested in the instantaneous pain relief provided by the packs, rather than any sustained anti-inflammatory response. We took a conservative approach to the timing here using the 60s cut off. Despite the conservative cut-off, we still observed differences in responses. Whilst anecdotal, we know from personal experience that the pain-relieving effect of cold to a burn wound is instantaneous, so it is fair to assume if a similar response in calves exists, it certainly will occur within that time-frame.

6.) Additional points that I would benefit from clarification on:
o I am struggling with some of the mismatch in language used throughout the paper, particularly when discussing different metrics to assess calf welfare. For example, in ln 51, it is said that behavioral and physiological evidence provide weak evidence for animal’s emotional state, but later in the paper, there seems to be strong conclusions drawn using behavioral metrics. e.g., ln 299-300 conclusions about these responses and how they link to emotional state are made.

>>> Sorry for the confusion, we meant ‘spontaneous behaviours’ (e.g., ear flicking) here. The sentence now reads (line 51-52): “spontaneous behaviours and physiological evidence provide weak…”

o There seems to be a discrepancy in the amount of discussion among results, where some considerations that are only tangential given much more space than those that are concrete and connected to results shown. For example, there a limited amount of discussion on the effects of milk allowance (ln 300-303; this is a concrete, important consideration to understand the findings), but other components that were not reported in this study, such as personality, were discussed in length (ln 333-341).

>>> We feel that the discussion around individual personality is important in the interpretation of our results, as explained in the discussion. We have however added to the discussion around diminishing milk volume. The paragraph now reads (line 328-337):

“The finding of a non-significant tendency for calves to come faster, as expected milk volume decreased, to the bucket associated with the cool pack but not to that associated with the ambient pack agrees with this observation, suggesting that the temperature of the pack affected the motivational trade-off between touch aversion and milk reward. However it is unclear whether this difference in behaviour is a result of time (and therefore subsidence of analgesia) or diminishing reward, and so further investigation is required. Interestingly, an initial rise in latency to the ambient pack within the first 24 h post-disbudding was not observed for the cool treatment, pointing to a potential benefit of cold therapy also within the first 24 h post-disbudding. These findings support our prediction that application of cold to wound sites post-disbudding provides pain relief.”

o Ln 109- 111: How was temperature maintained for the cold packs? Including this information would be helpful for others aiming to replicate your work

>>> Cold packs were kept in a fridge, maintained at 4ᵒC until use. Each pack was swapped for another, identical pack at 4ᵒC after each experimental session with a calf. Using an infrared thermometer, we confirmed that over the duration of a session (a maximum of 20 minutes), cool pack temperatures only increased by a maximum of 1ᵒC .

To clarify, we have added a statement to this effect (line 127-129): “Cool pack temperatures were monitored using an infrared thermometer (SOVARCATE HS960D), confirming that pack temperatures did not change by more than 1ᵒC during a given session.”

o Ln 146-147: Consider including details of the disbudding process, for example, the iron used and if the bud was left in or scooped out

>>> We have included the hot iron brand used, as well as the detail that the horn buds were scooped by the veterinarian. the section in ‘Baseline and disbudding’ now reads (line 169-171): “Hot-iron disbudding was performed using a hand-held gas iron (Portasol Gas Dehorner, Ritchey) immediately following session 5 by a trained veterinarian, with horn buds scooped out.”

o Ln 162-163: Could you provide an operational definition for “gazing” as part of your definition for latency to drink? How is this assessed for an animal with approximately 270 degrees in their visual field?

>>> We meant that the calves’ head was directed towards the open door. We established in pilot testing that in every instance before entering the test pen, calves would turn their head directly towards the door and look at the test pen. This was taken as the moment gazing commenced. A more specific measure of when calves first direct their attention to the open door is not practically possible. The sentence now reads (line 186-187):

“Latency to drink (herein, ‘latency’) was recorded using a stopwatch from the moment the calf in the lobby turned its head and directed its gaze towards the open door, until suckling commenced.”

o Ln 199-201: Why were the data transformed through the addition of a constant to eliminate negative values? Given the interest in change from baseline, wouldn’t the negative values have meaning?

>>> Gamma distributions cannot be fit to ≤0 values. The addition of the constant allows to use a gamma distribution (rather than use a less well-fitting normal distribution). The models are only used for assessing significance, and the addition of the constant does not affect the model predictions nor significance tests. negative values are still demonstrated in the graphical analysis.

o Ln 56-58: What is the benefit of phrasing the study results in this way as opposed to contextualizing CPP, which is a component of your research?

>>> in order to contextualise the CPP methodology in this paragraph, we have amended this section. it now reads (line 55-61):

“Conditioned place preference (CPP) tests – a commonly used paradigm to assess the rewarding effects of a specific stimulus or experience as a result of prior conditioning [28] – has also indicated that post-procedural pain and its associated emotional impact is remembered by calves [7]. Using the CPP paradigm, calves have also shown preference for the provision of analgesia with disbudding [29], as well as prolonged pain control (i.e., in the form of a local anaesthetic injection) up to 20 days after disbudding when compared to sham disbudded controls [14], suggesting they were still in pain.”

o Ln 156-160: Why choose to conduct the CPP at the end of testing sessions when milk has been decreased rather than after session 7? Could this affect the association the calves would have made in the sessions running up to the CPP?

>>> This is a good point. We did this to not jeopardize the learning for the approach-avoidance task. We are now adding this limitation in the discussion. We have included in the discussion the following (line 359-362):

“An additional limitation was running the CPP test following the completion of the approach-aversion experiment, after sessions where milk reward had decreased. We chose not to conduct CPP prior to reward diminution, so as to not jeopardize the learning for the approach- aversion task. This may also explain why we did not detect differences in the CPP test.”

o Ln 87-88: Did you investigate any effects of breed?

>>> We did not investigate the effects of breed as sample size did not allow it and this was not the aim of the study.

o Ln 122-123: Was the side of the pen the color was on also altered? Or was blue always left as depicted in Figure 1?

>>> The sides of the pen always remained the same. These were positioned for calves to better distinguish the left and right side of the pen from memory. The colours are therefore a proxy for left vs right side of the experimental pen.

to clarify this, we have amended the caption for figure 1 (line 119-122):

“(A) aerial diagram of test pen and adjoining lobby area with positioning of doors and milk buckets (to scale); (B) three-dimensional projection of test pens and lobby area (not to scale). The coloured boards were fixed to the pen walls, positioned for calves to better distinguish the left and right side of the pen from memory. The colours are therefore a proxy for left vs right side of the experimental pen.”

**Further comments from the Authors:**

>>> We have also taken the decision to change the colour on the graphs found in figure 3, as on reflection we feel that the colour combination detracted from the main aspects of the graph. The graphs now have a greyscale background, as below:
