# Supplementary material for: Cold therapy and pain relief after hot-iron disbudding in dairy calves
Source: PLoS One. 2024 Jul 12;19(7):e0306889. doi: 10.1371/journal.pone.0306889 (PMC11244798; doi:10.1371/journal.pone.0306889)
Supplement: Attachment — Submitted filename: Response to Reviewers.docx [file pone.0306889.s007.docx]

**Reviewer #1:** Thanks for your responses to the comments. I understand in science, it is sometimes necessary to "push" the boundaries; however, the applicability of this is limited. This should be discussed especially when producers aren't even doing "easy" methods of pain control (i.e. local + NSAID)
>>> We agree that the current practicalities of applying this research are difficult given the context. We have added the following to the end of the discussion to reflect this.

“With the current low levels of NSAID use on farms during disbudding procedures [17], additional research is also required before cold therapy can be practically applied on farms.”

Line 19: Please add in the duration of the contact time
>>> We are unable to provide an exact timing here as the timing varies between each calf and session.

Line 95 to 97: I find this revised sentence confusing. I would delete that other studies have used 15 calves previously as it doesn't add meaning

>>> We agree and we have therefore decided to remove the sentence completely.

**Reviewer #3:** Thanks to the authors for including the new figure, refining the use and predictions associated with the approach-aversion methodology and clarifying the conclusions about the new information generated about cold therapy. I have one outstanding concern in the R2 version.

I find the approach to excluding data (latency exceeded 30s, drinking exceeded 60s) concerning, as written. I’ve pasted the responses to Reviewers 1 and 3 about this topic below. From what I understand thus far, I am concerned about the approach taken, to exclude these data, has on the conclusions drawn.

A priori or post-hoc decision. Firstly, for the trials ended if latency was greater than 30s and drinking events were capped at 60s, I think it is important to know if this decision was made a priori or post hoc. As the authors point out, these kinds of cutoffs are common when running trial-based experiments. However, if these data were actually collected, then excluding them and imposing a 30s or 60s end was a post-hoc choice, this is worrisome to me. From my perspective, trimming a dataset to remove extreme values post hoc is not best practice.

>>> Apologies for the lack of clarity here. In both cases, this was an *a priori* decision.

For latency to enter the pen:

We apologise that we didn’t make this clearer, we did not collect values past cutoffs. Limiting latency to 30s cutoff was necessary given the time constraints we had during the experiment. As a result, we do not have actual extreme values, and 30 seconds represented a ceiling value.

For drinking duration:

We apologise for the lack of clarity. A trial was ended when a calf stopped drinking before finishing the milk reward, and did not recommence drinking within 60 seconds. This is for a similar reason to the 30s latency above and was also *a priori* - we only recorded the ceiling value of 60s in this case.

To emphasise the *a priori* nature of these decisions, we have amended the data collection section (lines 180-183, 189-191). It now reads:

“We took the *a priori* decision that, if a calf took more than 30s to enter the pen and contact the bottle, this was interpreted as a ‘no-go’ response, ending the trial. The calf would then be returned to the lobby area to commence the next trial, consistent with previous studies [27].”

and:

“If the calf stopped drinking before the milk bucket was empty, calves were given one minute to reinitiate drinking, otherwise the trial ended, with a 60 second ceiling value recorded (27/594 trials, 14/27 of which were ambient trials)”

Alternatives to exclusion. I appreciate that extreme values can affect statistical models, but exclusion should be a last resort. It is entirely possible that these responses represent an important part of the biological response of the calf. As the authors note, these represent “extremely aversive calf responses” and this was part of the question being asked. As such, exclusion is not “taking a conservative approach” as the authors describe it, at least from my perspective it is not conservative. To me, there are several options that would be preferable to the current approach of complete exclusion from a given model. Even when thresholds are set a priori, I prefer to enter a ceiling value for animals that do not meet or exceed a predetermined threshold, for example 31s for latency and 61s for drinking events, in this case (1 s beyond the cutoff). This approach allows the ceiling effect to be included while still meeting some of the assumptions of the statistical tests (e.g. normality). Another option, especially if data were collected beyond the thresholds (that is the decision about thresholds were post hoc) and actual ceilings were reached (e.g. say 80s drinking event), would be to keep all values in the dataset and explore data transformation in parametric models or use of non-parametric tests to address the model fit concerns the authors describe. Lastly, another approach I admire is to present the model outcomes with and without outliers (or extreme values in this case) included. This allows the reader to see their effect for themselves and make their own decision about how much they trust the findings.

>>> We thank the reviewer for these suggestions. As mentioned above, we do not have data collected beyond the thresholds due to the *a priori* nature of these decisions. Trials which ended due to these cut-offs do not have corresponding values for the ‘post-drinking contact duration’ or ‘nipple contacts’ metric, hence the ‘NAN’ values in the supplementary raw data file. To make that clearer we have added (lines 197-199):

“The number of nipple contacts or post-drinking contact duration were not recorded for trials which ended due to latency exceeding 30s or pauses in milk reward consumption exceeding 60s.”

In light of the reviewers’ concerns, we have amended our statistical analysis to include 30 second ceiling values (n=15) in the latency model and 60s ceiling values for drinking duration (n=27).

- For drinking duration, the inclusion of the 60s ceiling values had very little influence on post-disbudding statistics but was significant prior to disbudding. The t and p values have been amended accordingly to reflect the new model (lines 260-266).The pre-disbudding section now reads:

“Prior to disbudding, treatment pack had no influence on latency (t = -1.352, P = 0.187), post-drinking contact duration (t = -1.745, P = 0.086) or nipple contacts (t = -0.117, P = 0.907), but did influence drinking duration (estimate ± SE = 0.09 ± 0.05 s, t = 2.048, P = 0.041), suggesting calves took longer to finish drinking the milk reward with the cool pack prior to disbudding..”

We also reran the permutation statistic regarding the emptying rates of the buckets on each side of the area. With the inclusion of the 60s ceiling values (n=27), there is no longer a statistically significant difference between the bucket emptying rates. This section has now been edited to reflect this (lines 292-294):

“On average, the two buckets were not found to empty at different rates (permutation test, P = 0.088), suggesting that the emptying rates of the two buckets does not help to explain the differences in colour.”

- For Latency, regarding the 30s ceiling values for the Latency post-disbudding model, we particularly like the suggestion of providing statistical metrics for both models, with and without these 30s ceiling values. We have amended our methods such that the model that includes the 30s ceiling values is now the default model
  (with p and t values amended to reflect this – line 272). We have also removed the sentences relating to the interaction term (p=0.071) in the post disbudding results section and discussion section, as the new model does not produce such p values of note, and replaced it with the following results (lines 279-281):

“Furthermore, no significant interaction effect between milk volume and treatment was observed for latency (P = 0.573), drinking duration (P = 0.713), post-drinking contact duration (P = 0.846) or nipple contacts (P = 0.831).”

The second model that excludes these *a priori* ceiling values (as in the original manuscript) has been included as supplementary material as it improved the model fit, allowing readers to draw their own conclusions regarding the two models. This second model has been referred to in the methods section on (lines 238-241):

“For latency, a second post-disbudding GLMM was also constructed, which excluded the 30 second ceiling values due to concerns about the their influence on model fit and interpretability. This model better fit the latency data with regards to AIC/BIC criteria and Q-Q plots (see supplementary table (S4)).”

and in the results section on (lines 273-275):

“The influence of pack temperature on changes in latency was still observed when 30 second ceiling values were excluded from the analysis (t = -2.11, 95% CI: -0.181, -0.007, P = 0.035; Fig. S4).”

The raw data file has also been amended to include the 30s/60s ceiling values, and will be reuploaded on resubmission.

Treatment differences. Lastly, the treatment distribution of the excluded values (or even if ceiling values are entered instead, as suggested above) should be described. I did not see a response to this aspect of my question in the authors’ response.

>>> We apologise for missing this aspect of your previous question. We have added a breakdown of the treatment differences in the data collection section (lines 184-185)

For latency: “...for subsequent statistical analysis (15/594 trials, 7/15 of which were ambient trials).”

For drinking duration: “...with a 60 second ceiling value recorded (27/594 trials, 14/27 of which were ambient trials”.

We should also add, the number of latency trials recorded as 30s was recorded erroneously as 37 in the previous version (as it included trials for testing/habituation phase which were never meant for inclusion in the statistical analysis). It is now correctly displayed as 15 trials. A further 6/594 trials had latencies recorded as ‘nan’due to technical recording difficulties during the trial, as so were excluded. Previously, ratios as above were given as out of 588 (to account for the 6 excluded trials), but we have made these 6 exclusions more explicit, giving ratios out of 594 instead and including a comment about the 6 excluded trials. This additional detail has been added to the results on (lines 269-270):

“Six trials were excluded following data collection due to technical issues, resulting in a total of 588 trials across all individuals.”

We would also like to note that we have amended slightly the plots in figure 3, as the previous submission had an error (the title for plot B had ‘A)’ in the title by mistake. This has been amended and reuploaded. The uploaded plot for Latency shows the graph with 30s ceiling latencies included.
